# Operator-guided Navigator Gating for Real-Time Interactive Coronary Cardiovascular Magnetic Resonance

**DOI:** 10.1186/1532-429X-18-S1-P321

**Published:** 2016-01-27

**Authors:** Keigo Kawaji, Mita Patel, Jouke Smink, Hui Wang, Roberto Lang, Amit R Patel

**Affiliations:** 1 Medicine, Section of Cardiology, The University of Chicago, Chicago, IL USA; 2 Philips Healthcare, Best, Netherlands; 3 Philips Healthcare, Cleveland, OH USA

## Background

Real-time interactive (RTI) MRI parameter manipulation during the scan [1] may potentially address challenges imposed by respiratory motion during a free-breathing cardiovascular magnetic resonance (CMR) acquisition. In this study, we propose an operator-guided processing that allows manipulation of navigator gating parameters in real-time. This approach was evaluated in healthy volunteers, where coronary CMR (CCMR) with and without RTI manipulation was examined to assess acquisition failure rates, scan time reduction, and vessel sharpness.

## Methods

**Figure 1:**
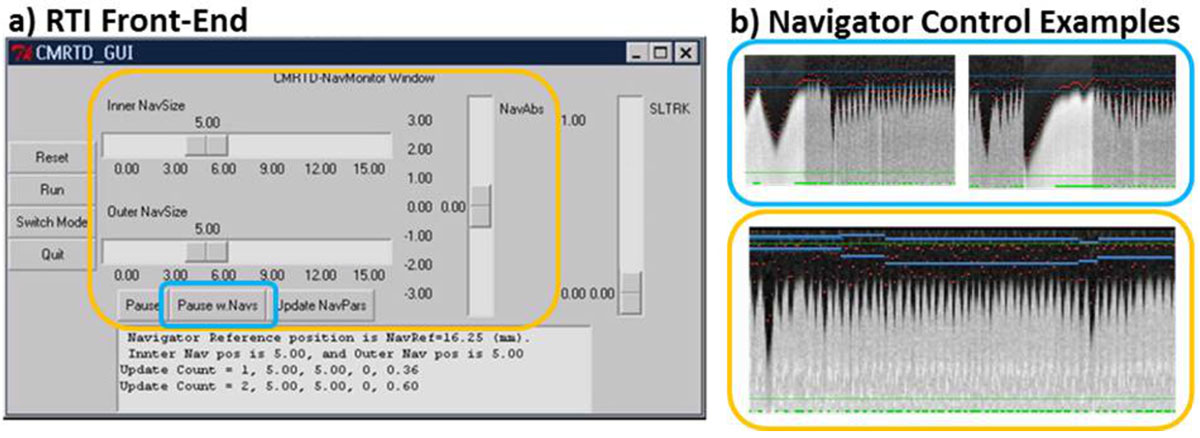
**Real-time and Interactive control of navigator parameters**. a) Graphical User Interface. b) Operator control examples - blue: of manual pause and repeated navigator during breath-hold instruction (used in n = 1 subject in this study); orange: interactive manipulation of navigator gating window position parameters (used in all 13 subjects). This example shows the operator manually adjusting the gating window to the subject's respiratory drift position.

The proposed RTI framework employs a custom communication protocol between the scanner host and the waveform generation hardware that allows non-time-critical operator tasks (ie. made changes are reflected in the next collected heart beat without overtasking the scan runtime). The custom front-end (Figure 1a) shows the operator-interactive navigator control that allows manipulation of the diaphragm navigator gating window in real-time (Figure 1b).

This RTI approach was incorporated into a CCMR sequence with view/profile order compatible with weighted navigator gating. 13 healthy volunteers were imaged on a 1.5T system (Philips Achieva) using a 5 channel cardiac array. Scan parameters were: TR = 4.4 ms; TE = 1.9 ms; FA = 90; 300 × 300 × 100-130 mm^3^ at 1.3 mm^3^, interpolated to 0.65 × 0.65 × 1.3 mm resolution; Sensitive Encoding (R = 2) was used. The default gating window was 5 mm set by a 20-heartbeat (HB) calibration. Slice tracking was not used. Two volumes were acquired; one employing RTI, and another without using this tool. RTI and non-RTI CCMR acquisitions were randomized. Total number of HBs (calibration HBs not included), navigator efficiency (NavEff), and vessel sharpness in the RCA, LAD, and LCX were measured. Student's t-test was used for statistical analysis.

## Results

**Figure 2:**
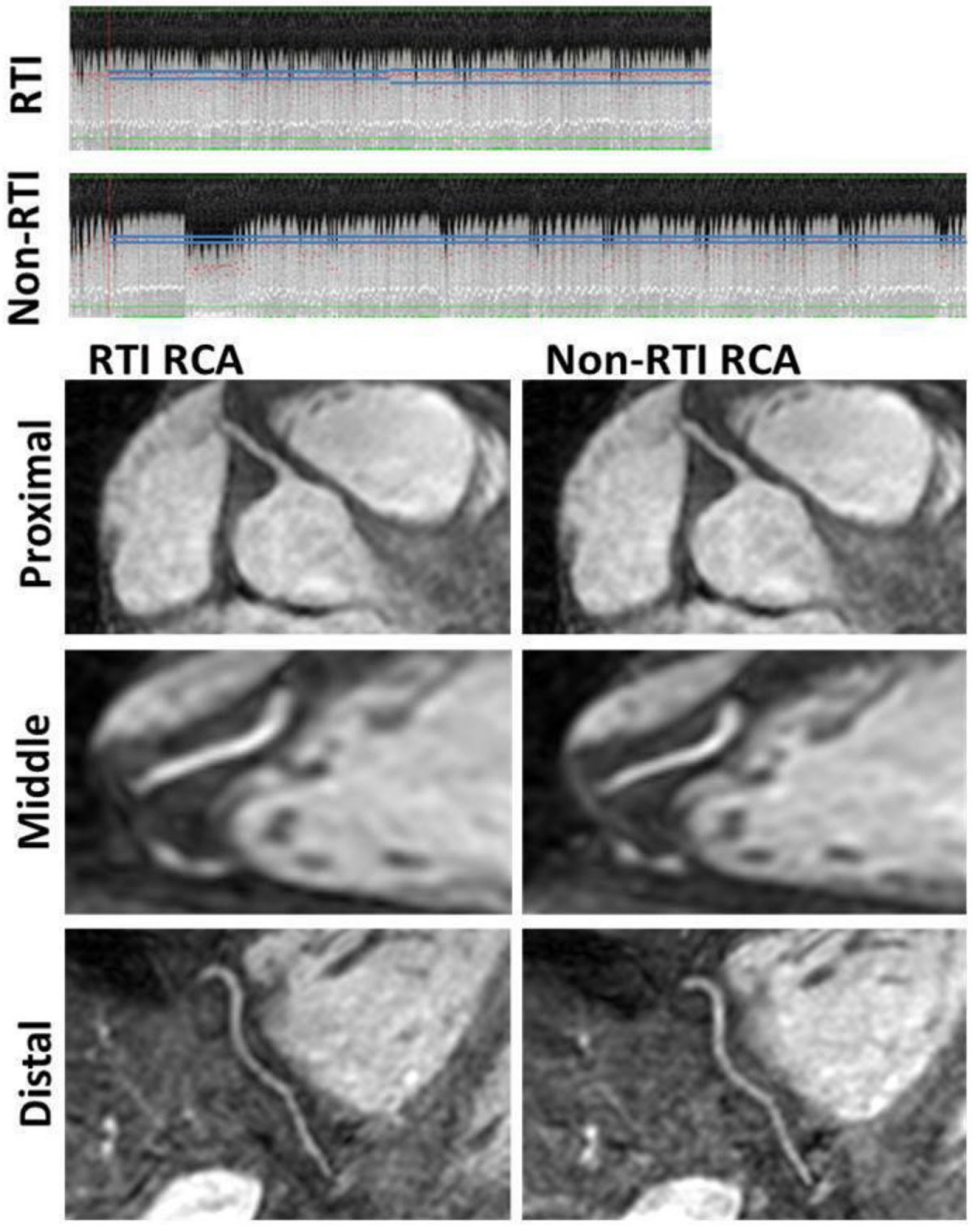
**Example of RTI adjusted CCMR compared against a non-RTI approach reference**. Top: RTI and non-RTI NAV profiles. RTI: 324 HBs; NavEff = 62% vs non-RTI: 438 HBs and 46%. Operator interaction involved expanding the navigator gating window size from 5 mm to 7.5 mm midway (136th out of 324 HBs) during the scan. This increased the navigator efficiency from 56% (first 136 HBs) to 66% (Remainig 188 HBs). Bottom: Acquired RCAs. Sharpness scores were (RTI: 0.52) vs (non-RTI: 0.48).

The proposed RTI tool allowed successful completion of 3D coronary acquisition in all 13 subjects (375 ± 67 HBs, NavEff = 56 ± 9%). Figure 2 shows a representative example. The non-RTI scans resulted in the operator restarting the scan in seven subjects (n = 8 total restarts; stopped @ 82 ± 51 HBs w. NavEff = 26 ± 12%; restart rate = 40% [8/20 scans]). Of these, non-RTI data was not collected in n = 1 due to significant respiratory drifting. The total HBs for n = 12 non-RTI scans were 443 ± 76 (p < 0.001 vs RTI), with NavEff = 48 ± 6% (p < 0.005 vs RTI). Sharpness scores (RTI vs non-RTI) were as follows: RCA (0.48 ± 0.04 vs 0.46 ± 0.05; p < 0.05), LAD (0.41 ± 0.06 vs 0.42 ± 0.04; p=NS), and LCX (0.40 ± 0.05 vs 0.41 ± 0.04; p=NS).

## Conclusions

The feasibility of RTI manipulation between waveform generator and host console during MRI data acquisition was successfully demonstrated without need for additional dedicated research hardware. RTI operator-guided manipulation of the navigator gating window eliminated repeated acquisitions of 3D CCMR sequences in all 13 subjects, while achieving ~70 fewer HBs, ~8% NavEff increase, and improved/comparable sharpness compared to conventional non-RTI CCMRs.
